# Tunable fractional Fourier transform implementation of electronic wave functions in atomically thin materials

**DOI:** 10.3762/bjnano.9.174

**Published:** 2018-06-19

**Authors:** Daniela Dragoman

**Affiliations:** 1 University of Bucharest, Physics Faculty, P.O. Box MG-11, 077125 Bucharest, Romania; 2 Academy of Romanian Scientists, Splaiul Independentei 54, 050094, Bucharest, Romania

**Keywords:** atomically thin materials, Fourier transform, tunable devices

## Abstract

A tunable fractional Fourier transform of the quantum wave function of electrons satisfying either the Schrödinger or the Dirac equation can be implemented in an atomically thin material by a parabolic potential distribution applied on a direction transverse to that of electron propagation. The difference between the propagation lengths necessary to obtain a fractional Fourier transform of a given order in these two cases could be seen as a manifestation of the Berry phase. The Fourier transform of the electron wave function is a particular case of the fractional Fourier transform. If the input and output wave functions are discretized, this configuration implements in one step the discrete fractional Fourier transform, in particular the discrete Fourier transform, and thus can act as a coprocessor in integrated logic circuits.

## Introduction

Despite continuous advances in nanotechnology, existing Boolean computer architectures based on CMOS technology are reaching their limits of integration that emerge due to several problems, including those related to the increase in the number of interconnects and to heat dissipation [1–2]. Several alternatives, such as quantum computing [3], have been proposed and even realized with superconducting qubits [4], but no affordable, room-temperature substitute to present-day desktop computers is available up to now.

On the other hand, the development of enhanced computing architectures fabricated with the help of the current nanotechnology could alleviate at least some problems associated with classical computing circuits, in particular with the irreversible nature of their logic operations. For instance, logic architectures employing reversible gates based on an as small as possible number of elements/transistors could minimize the heat dissipation problem. Indeed, several such logic gate configurations in graphene have been recently proposed [5].

Another efficient way to increase the computation speed and integration scale of computing circuits would be to design configurations able to implement logic algorithms in a direct way, without decomposing them into constituent logic gates. For example, it was recently shown that the quantum Deutsch–Jozsa algorithm could be implemented in one step, in a relatively simple graphene-based configuration, and not using a succession of quantum gates [6].

In this paper we propose a configuration that can implement in a direct way/in one step an integral transform of a quantum wave function of ballistic electrons, namely the fractional Fourier transform (FrFT); this configuration is in fact a FrFT coprocessor. In particular, the Fourier transform, which is a special case of the FrFT, is of interest in digital signal processing [7] as well as in many quantum computing algorithms [8], via the discrete Fourier transform. Moreover, the proposed configuration allows the implementation of a FrFT with a tunable order in atomically thin materials in which the charge carriers obey either the Schrödinger equation (as in two-dimensional electron gases (2DEGs)) or a Dirac-like equation (as in graphene). The difference between these two cases, expressed by a different relation between the propagation length and the FrFT order, reveals at the same time another manifestation, as yet unexplored, of the Berry phase in graphene [9]. The configuration proposed for FrFT implementation requires a ballistic transport regime of charge carriers. Graphene could be of particular interest for this application because it has the longest, micrometer-scale, room-temperature mean free path of all known materials [10]. The proposed implementation method of the FrFT of electron wave functions is based on the analogy between the classical electromagnetic field and the quantum wave function of ballistic electrons [11], as well as on the fact that the FrFT in optics can be implemented in graded index (GRIN) waveguides with a refractive index that varies quadratically with the transverse coordinate [12–13]. Because, unlike in [14], the configuration implementing the FrFT of quantum wave functions proposed in this paper works for both 2DEGs and graphene, and does not require magnetic fields, it is much more suitable for integration in logic circuits.

## Results

The Fourier transfom of a function ψ(*x*) is a particular case, corresponding to α = π/2, of the FrFT of order α, defined as [12]

[1]
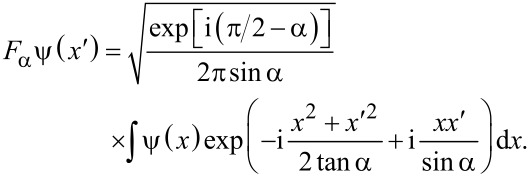


As mentioned already, we rely on the analogy between the classical electromagnetic field and the quantum wave function of ballistic electrons satisfying the Schrödinger equation [11] to implement the FrFT of electron wave functions. In optics, the FrFT of order α of an incident optical field *E*(*x*,*y*) = *E*(*x*)exp(i*ky*), which propagates with a propagation constant *k* along the *y* direction in a planar GRIN waveguide with refractive index *n*(*x*) = *n*_0_ − *n*_1_*x*^2^/2, and satisfies the Helmholtz equation

[2]
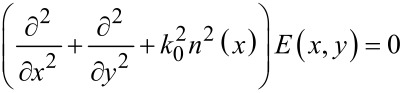


is obtained after a propagation distance 
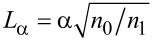
 [12–13]. In Equation 2, *k*_0_ = ω/*c*, where ω is the frequency of the electromagnetic wave and, in general, the second term in the refractive index expression is much smaller than the first one. As detailed in [12], this result follows from identifying the discrete eigenvalues of FrFT and of Equation 2, the eigenmodes in both cases being Hermite–Gauss functions.

Consider now an atomically thin material in which the quantum wave function of ballistic electrons with energy *E* and effective mass *m*, Ψ(*x*,*y*) = ψ(*x*)exp(iκ*y*), propagates along the *y* direction, with a propagation constant κ, and satisfies the time-independent Schrödinger equation

[3]
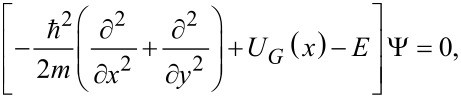


where the electrostatic potential energy *U_G_*(*x*) is induced via a gate voltage. We refer in the following to such a material as a 2DEG, in order to distinguish it from graphene, an atomically thin material in which electrons obey a different equation. Due to the similarity of Equation 2 and Equation 3, a FrFT could be implemented if the electrostatic potential depends quadratically on the transverse variable *x*: *U_G_*(*x*) = *U*_0_ + γ*x*^2^, where the second term can be considered as a perturbation of the first one. Indeed, following the procedure indicated in [8], or simply observing that Equation 3 is identical to Equation 2 if the following substitutions are made: 
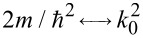
, 
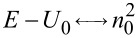
, 
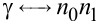
, we find that a FrFT of order α of the ballistic wave function is obtained after propagating in the 2DEG along a distance

[4]
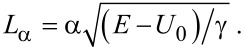


For a device with a given length, the order of the FrFT at the output can be tuned by varying the potential energy, i.e., the parameters *U*_0_ and γ.

The GRIN optical waveguide/electrostatic potential modulates periodically the optical field/quantum wave function, as can be seen from the matrix relating the transverse coordinate *x* and the tangent θ to the ray/quantum trajectory at a *y* = const. plane and the same parameters (indexed by 0) at the *y* = 0 plane [15]:

[5]
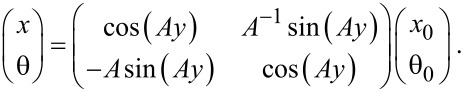


In Equation 5, valid when 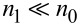 or 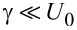, one has 
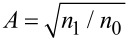
 in optics and 
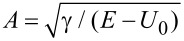
 in the case of Schrödinger electrons.

Whether the results above regarding Schrödinger electrons are not unexpected, the question is if the same configuration can be used to implement a FrFT of order α in graphene, where electrons satisfy a Dirac-like equation. In this case the wave function is spinorial: Ψ*^T^* = (ψ_1_,ψ_2_), and we consider electrons propagating along the *y* direction with a propagation constant κ, i.e., if Ψ(*x*,*y*) = Ψ(*x*)exp(iκ*y*), in the same potential energy distribution, such that the wave function is a solution of the equation

[6]
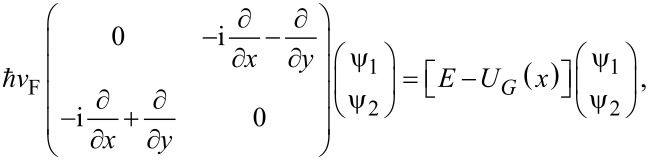


where *v*_F_ ≈ *c*/300 is the Fermi velocity. From Equation 6 it follows that, for slowly varying potential energy distributions, as assumed above, for which


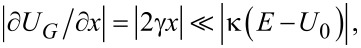


both components of the spinorial wave function satisfy the equation

[7]
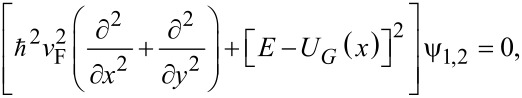


which is similar to Equation 2 if 
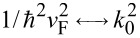
, 
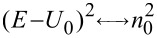
, 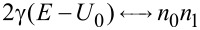. As a result, the FrFT of order α is achieved after a propagation distance in graphene equal to

[8]
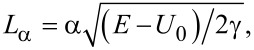


and the trajectory of charge carriers in graphene is also periodic and described by Equation 5, with 
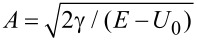
. Again, the order α for a given propagation length can be tuned by varying the potential distribution via a gate voltage.

It should be emphasized that *L*_α_ in Equation 8 is √2-times smaller than the propagation distance required for obtaining the same effect in 2DEGs with Schrödinger electrons. Despite the completely different equations satisfied by electrons in these latter materials and graphene, in which specific parameters such as electron mass and Fermi velocity appear, the propagation distances needed to implement the FrFT of an arbitrary order α differ by a constant factor only. This can be seen as another, up to now unidentified, manifestation of the Berry phase in graphene [9,16].

Please note that for both, 2DEGs and graphene, *L*_α_ does not depend on the wave-vector component along the *y* direction, but only on the energy and the parameters of the potential energy distribution. This means that the wave function does not need to be of plane-wave type, i.e., it can also be spatially divergent or convergent. In particular, divergent wave functions, as in [17], can be produced by localized electron sources/sharp tunneling conductive tips. In general, the shape of the transverse wave function can be tuned via boundary conditions by patterned electrodes and/or gate electrodes, such as encountered in 2DEG electron-optics experiments (see [18] for a review). Alternatively, nonintentional potential inhomogeneities within the sample can influence the spatial distribution of the wave function. On the other hand, electron wave function propagation along the *y* direction can be easily assured by applying a bias along *y*.

It should be emphasized that configurations that implement FrFTs or discrete Fourier transforms of quantum wave functions based on the analogy with the classical electromagnetic field have been proposed already. For instance, it was shown that a tunable continuous FrFT of the wave function of a 2DEG can be obtained by applying an in-plane magnetic field [14], while a discrete Fourier transform based on an array of four quantum waveguides was demonstrated in [19]. The advantage of the proposal put forward in this paper is that, besides the fact that it works for both 2DEGs and graphene, it does not require magnetic fields, being thus more suited for integration in nanoelectronics circuits, and that it implements a tunable FrFT that could eventually be discretized in more than four input or output entries. In addition, another attempt to implement discrete Fourier transforms in nanostructures [20] uses ring- or peak-shaped electrically controllable 2D potentials that act as scattering centers, but the actual implementation of these potential distributions is much more difficult to be achieved than the smoothly varying parabolic potential distribution proposed in this paper.

## Discussion

**Figure 1:**
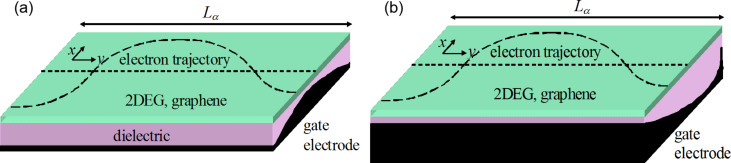
Schematic representation of a configuration that implements a tunable FrFT using (a) a convex, and (b) a concave gate electrode.

A parabolic distribution of the electrostatic potential energy could be obtained with a nonplanar, convex or concave gate electrode, as those represented in Figure 1.

Electrode arrays with convex or concave geometries have been fabricated on flexible substrates for the purpose of acting as brain–computer interfaces [21], while single nanoscale nonplanar electrodes with complex topographies have been obtained using advanced stencil lithography [22].

Because γ > 0, the configuration illustrated in Figure 1a implements a FrFT for negative *U_G_* potentials, while that in Figure 1b implements it for positive *U_G_* values. Figure 1a and Figure 1b depict also the trajectory of a ballistic electron. From Equation 4 and Equation 8 it follows that the last configuration implements a FrFT with a given α in a shorter distance, and should thus be used to fabricate devices with a smaller length, shorter than the mean free path, with graphene achieving the same task in a √2-times shorter distance than a 2DEG. In particular, if we want to implement a Fourier transform in a 2DEG in a distance of *L*_π/2_, the parameters *U*_0_ and γ of the quadratic potential distribution must be related to the electron energy as (*E* − *U*_0_)/γ = (2*L*_π/2_/π)^2^. Small values of *L*_π/2_ and of γ require that the electron energy is very close to *U*_0_, the latter parameter being tunable via the gate potential.

**Figure 2:**
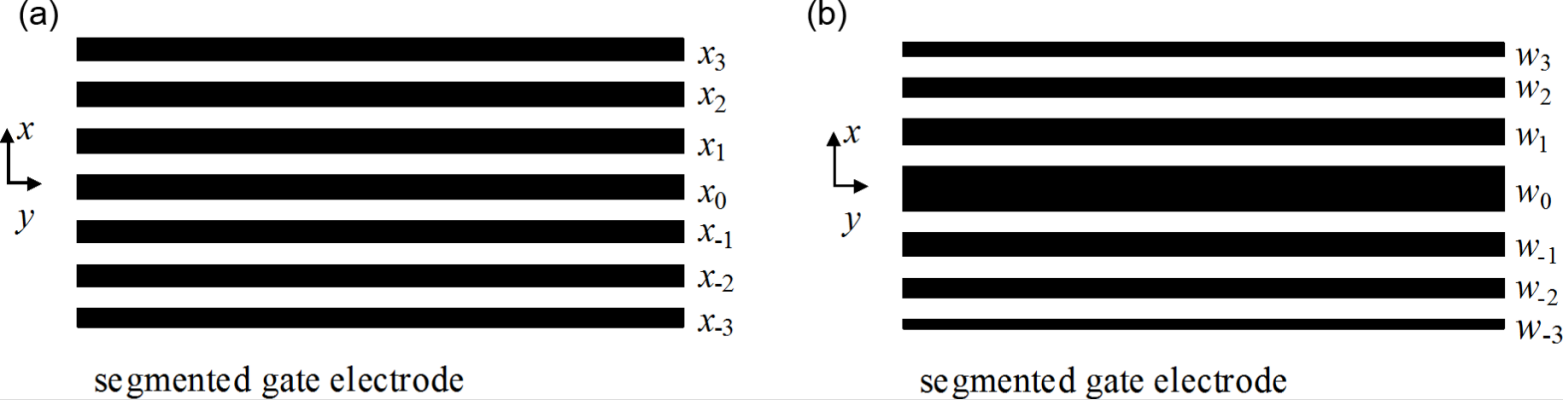
Schematic representation of a segmented gate electrode with segments with (a) equal and (b) unequal widths.

Although nonplanar electrodes could be fabricated, a simpler technological solution to achieve a parabolic potential energy distribution relies on segmented gate electrodes, composed of several metallic stripes with identical or different widths, as shown in Figure 2a and Figure 2b, respectively; the distance between electrodes, *d*, is assumed constant.

In the first case the metallic electrodes with the same widths, *w*, should be contacted independently, and the gate potential applied on the *n-*th segment, the center of which has the coordinate *x_n_* should be chosen such that

[9]
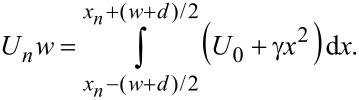


In the second case, a parabolic potential energy can be induced by a segmented gate electrode for which all segments are connected at/subject to the same gate potential. If the induced energy potential is *U*, the width of the *n*-th segment, *w_n_*, the center of which has the coordinate *x_n_*, should be chosen such that

[10]
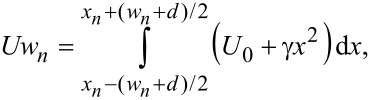


where *x_n_* satisfies the relation *x_n_* = *x_n_*_−1_ + *d* + (*w_n_*_−1_ + *w_n_*)/2. Equation 9 and Equation 10 express the condition that the average potential energy is the same in a segmented and in a continuous electrode, similar to the condition imposed in optics for a binary implementation of a parabolic refractive index profile [23].

The degree of approximation of a continuously varying potential with a piece-wise one depends on the number of segments in the electrode. Assuming that the minimum feature that can be attained is 30 nm, a period *d* + *w* should be at least 60 nm in width, so that, in order to have an electrode with seven segments in the configuration represented in Figure 2a, the width of the atomically thin material should be about 400 nm. Such transverse dimensions are attainable at least in the case of graphene.

In all situations, at the end of a 2DEG or graphene of length *L* the order of the implemented FrFT can be varied via the applied gate voltage, as indicated by Equation 4 and Equation 8. In particular, the Fourier transform can be obtained if *L* = *L*_π/2_.

If measurable, a tunable FrFT could be used to gather information on the quantum wave function of charge carriers. In atomically thin materials [23–24], as well as in graphene [25], only mappings of the probability distribution of charge carriers can be obtained using a scanning probe microscope. These (*x*,*y*) mappings correspond to different values of α along the *y* direction, and thus implement the α-tunable Radon transform 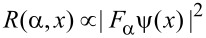 of the incident quantum wave functions, which is known to be able to fully recover the classical or quantum input signal via phase-space tomography [26–28]. As such, the quantum wave function could be recovered, albeit not directly measured.

It should be noted that the configuration described in this paper can also be employed to widen or narrow the electron distribution if it is set such that *L* = *L*_π/2_. Indeed, the Fourier transform of a wide/narrow signal/quantum wave function is narrow/wide, the width of the electron distribution being thus modulated by a Fourier transform device. Generally, for a device that implements the FrFT of order α of an input Gaussian distribution/wave function of width ξ_0_, i.e., 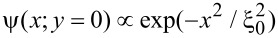, the width of the output wave function is [14]

[11]
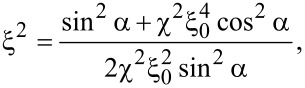


where 
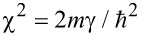
 for the 2DEG and 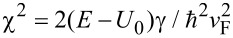 for graphene. These expressions, which can be derived also by performing the FrFT in Equation 1 show that the width of the outgoing electron distribution can be continuously modulated via gate voltages in the configuration proposed in this paper. This modulation could be of interest in ballistic electron devices.

**Figure 3:**
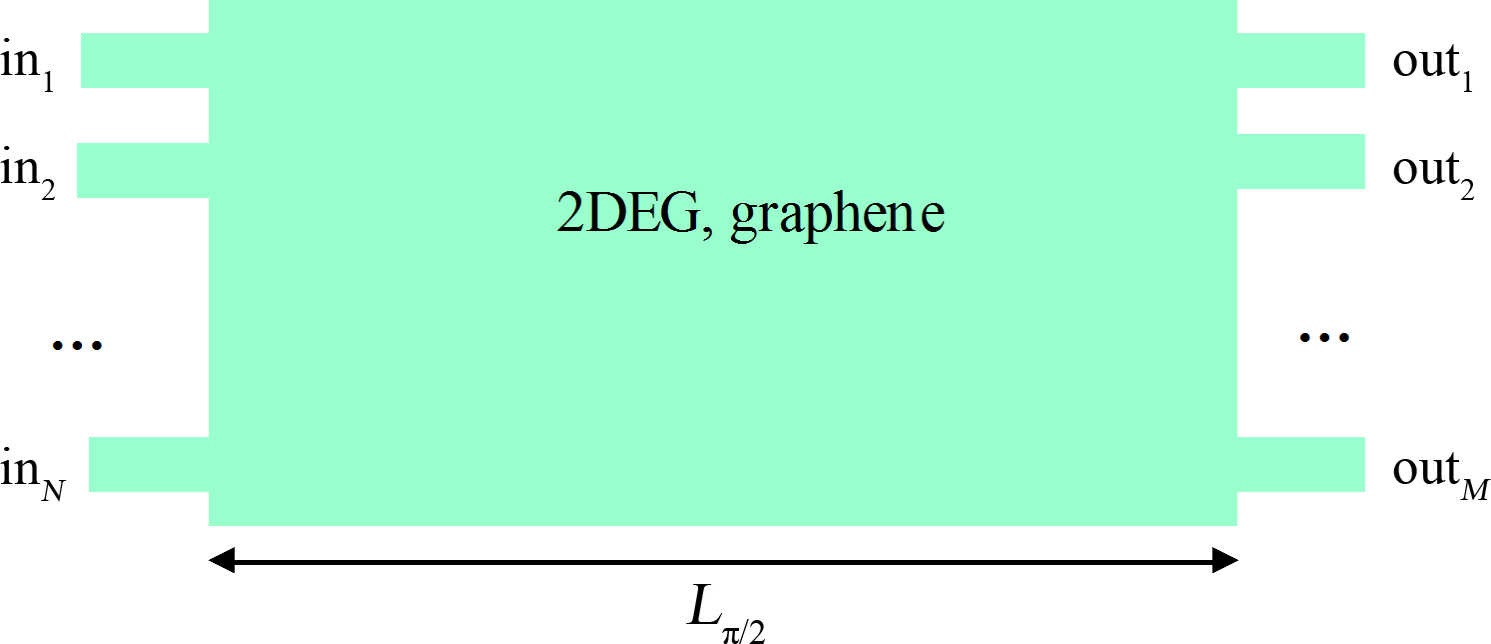
Schematic representation of a configuration that implements a discrete Fourier transform by discretizing the input and output channels of the device.

In another application, a series of narrow detectors could discretize the output in a number of, say, *M* channels. If the input is also discretized in *N* channels, the configuration proposed in this paper is able to implement a discrete FrFT, in particular a discrete Fourier transform (Figure 3) of a quantum wave function of an atomically thin material (2DEG or graphene). This configuration can then be used as a discrete FrFT or discrete Fourier transform coprocessor in integrated logic circuits. As mentioned already, the discrete Fourier transform is essential in digital signal processing [7] and computing algorithms [8], the discrete FrFT being not so much studied yet. However, it could become interesting in the future. It should be noted that discretized input and/or output channels could introduce significant scattering of the wave function, such that the retrieval of the FrFT (and/or of the unknown wave function) becomes more complex. Fortunately, a wide variety of techniques have been developed already to extract the relevant information from measurements of output currents/signals from waveguide or sensor arrays [29–31] (to mention only a few references). Indeed, the inverse scattering problem has extensive applications in everyday life, so that retrieval algorithms exist or can be adapted for virtually any situation.

## Conclusion

In conclusion, a parabolic potential distribution applied in a direction transverse to that of electron propagation in an atomically thin material implements in one step a tunable FrFT irrespective of the equation (Schrödinger or Dirac) satisfied by electrons. The difference between the propagation lengths necessary to obtain a FrFT of a given order in the two cases could be interpreted as a manifestation of the Berry phase. The Fourier transform of the quantum wave function of electrons is then obtained as a particular case of the FrFT. This configuration can be used to recover the quantum wave function via 2D mappings of the probability distribution, can modulate the width of the outgoing wave function and/or can act as a discrete FrFT, in particular as a discrete Fourier transform, if both the input and output wave functions are discretized in several entries. In the latter case the proposed configuration could be used as a coprocessor in integrated logic circuits.
